# Large deletions in immunoglobulin genes are associated with a sustained absence of DNA Polymerase η

**DOI:** 10.1038/s41598-020-58180-7

**Published:** 2020-01-28

**Authors:** Leticia K. Lerner, Thuy V. Nguyen, Ligia P. Castro, Juliana B. Vilar, Veridiana Munford, Morwenna Le Guillou, Mahwish Mian Mohammad, Véronique Vergé, Filippo Rosselli, Carlos F. M. Menck, Alain Sarasin, Said Aoufouchi

**Affiliations:** 1 Centre National de la Recherche Scientifique UMR8200, Gustave Roussy, 94805 Villejuif, France; 2 Department of Microbiology, Institute of Biomedical Sciences, University of São Paulo, São Paulo, Brazil; 3 Haematology Unit, Gustave Roussy, 94805 Villejuif, France; 4 Université Paris-Saclay, 91400 Orsay, France; 5 Sorbonne Université, Paris, 75006 France; 6 Present Address: Department of Genetics, Faculty of Biology, University of Science, Ho Chi Minh City, Vietnam

**Keywords:** Immunogenetics, Genetics research

## Abstract

Somatic hypermutation of immunoglobulin genes is a highly mutagenic process that is B cell-specific and occurs during antigen-driven responses leading to antigen specificity and antibody affinity maturation. Mutations at the Ig locus are initiated by Activation-Induced cytidine Deaminase and are equally distributed at G/C and A/T bases. This requires the establishment of error-prone repair pathways involving the activity of several low fidelity DNA polymerases. In the physiological context, the G/C base pair mutations involve multiple error-prone DNA polymerases, while the generation of mutations at A/T base pairs depends exclusively on the activity of DNA polymerase η. Using two large cohorts of individuals with xeroderma pigmentosum variant (XP-V), we report that the pattern of mutations at Ig genes becomes highly enriched with large deletions. This observation is more striking for patients older than 50 years. We propose that the absence of Pol η allows the recruitment of other DNA polymerases that profoundly affect the Ig genomic landscape.

## Introduction

Xeroderma pigmentosum (XP) is a rare, autosomal, recessive disease, characterized by photosensitivity, poikiloderma of sunlight-exposed areas appearing in childhood, and characterized by a 1,000- to 10,000-fold increase in the incidence of cancers in sun-exposed regions of the body (melanoma and non-melanoma skin cancers)^1^. Classical XP patients harbor mutations in one of the seven *XPA* to *XPG* genes^2^, which encode proteins that are part of the Nucleotide Excision Repair (NER) pathway involved in the removal of UV-induced cyclobutane pyrimidine dimers (CPDs) and (6-4) photoproducts (6-4 PPs)^3^. In contrast, patients with the XP variant (XP-V) form retain a normal NER pathway but carry on inactivating bi-allelic mutations in the *POLH* gene. XP-V patients (OMIM: 278750) account for approximately 20% of all XP patients worldwide. They exhibit some photosensitivity, usually after the age of 15, and may develop multiple skin carcinomas and melanomas with age. XP-V patients have milder and distinct clinical presentations compared to those found in the other XP complementation groups. In particular, they are characterized by: (i) a delayed cancer onset with tumors appearing in 20–30 years old individuals; (ii) variable severity, and (iii) lack of neurologic abnormalities^4–6^.

*POLH* codes for the DNA polymerase η (Pol η)^7,8^, a Y-family DNA polymerase specialized in the translesion synthesis (TLS) of CPDs^9^, a DNA lesion that blocks replicative polymerases. Following replication fork stalling, Pol η binds CPD-containing DNA with higher affinity than undamaged DNA, and incorporates dAMP efficiently past *cis-syn* thymine-thymine dimers^10^. In cells lacking Pol η, it is admitted that the bypass of CPDs is carried out by other TLS polymerases that are extremely more mutagenic, like pols ζ (*zeta*), κ (*kappa*) or ι (*iota*), which accounts for the occurrence of mutations and consequently for the increased incidence of sun-exposed skin cancers in XP-V patients^10^. Indeed, the analysis of somatic mutations found in skin cancers isolated from XP-V patients clearly indicates that the mutations are caused by misincorporation of nucleotides opposite unrepaired CPD lesions by another TLS polymerase than Pol η^11^.

Besides its established function in the error-free bypass of UV-induced lesions, Pol η is involved in immunoglobulin (Ig) gene somatic hypermutation (SHM), a unique and highly mutagenic process specific to activated B lymphocytes, which occurs in germinal centers (GC) of secondary lymphoid organs. The SHM process generates cells displaying receptors with the highest affinity and specificity for a given antigen^12^ that will differentiate into either plasmocytes or memory B cells^13^. SHM targets the variable regions of both the heavy and light chains of the Igs, mainly in a region of approximately 2 kb comprising the coding exons of the V(D)J genes and downstream the intron J_H_^14^.

Mutations are initiated by the activity of Activation-Induced cytidine Deaminase (AID) that converts cytosines to uracils^15^. The resulting U/G mismatch triggers an error-prone DNA repair in the Ig locus of the activated B cells. In collaboration with UNG, MSH2, MSH6, EXO1 and PCNA, mutations are generated during the processing of dU by several error-prone polymerases. On one hand, Rev1, Pol ι and Pol ζ induce mainly single and tandem mutations of C/G templates during S phase^16,17^, and, on the other hand, Pol η is responsible, in G1, for virtually all mutations at A/T sites^18–20^. Therefore, B-cells from XP-V patients present a decreased frequency of mutations in A/T sites and a bias towards G/C template mutations, even though the frequency of overall mutations reported is not strongly affected^21^. A similar phenotype was found in Pol η-deficient mice^18,19^.

We have previously described a direct correlation between Pol η activity and the frequency of A/T mutations, showing that one XP-V patient with reduced amounts of functional enzyme possesses an intermediate frequency of A/T mutations compared to XP-V patients that completely lack Pol η^21,22^. Furthermore, using mouse models, we have shown that, in the complete absence of Pol η, mismatch repair-dependent mutations are introduced by one or more DNA polymerases that are not usually involved in SHM. We have shown that at least another DNA polymerase, Pol κ, can be mobilized during SHM in the complete absence of Pol η leading to a new mutational signature. Indeed, we identified a Pol κ signature in an analyzed Pol η-deficiency patient^19,22^.

So far, most published data on SHM of Ig genes have been obtained from a restrained number of patients making it less robust and difficult to generalize. In this report, we have analyzed the pattern of Ig SHM in two large cohorts of XP-V patients aged13 to 85 years displaying different mutations in the *POLH* gene. We have shown that the A/T mutation pattern at the Ig gene accurately mirrors the extent of Pol η activity and therefore can be used in the clinic as a genuine and reliable assay for the XP-V diagnosis. Moreover, we noticed that, in the absence of Pol η, its substitution by other TLS polymerases leads to a modified landscape of mutations with an increase rate of deletions and insertions, especially in patients older than 50 years.

## Results

### Characteristics of XP-V patients and controls

In this work we analyzed the SHM profile in terms of levels and patterns, by sequencing a PCR-amplified segment in the J_H_4 intronic region from isolated memory B cells from XP-V patients and controls from two large XP-V cohorts, which we called French and Brazilian cohorts. Both cohorts are similar for age (Supplemental Fig. S1). Patients were classified, according to the severity of symptoms, into three groups of aggressive, medium, and mild symptoms^5^. This classification was made by specialized cancer clinicians and dermatologists taking into account the following criteria: skin abnormalities; age at diagnosis and at the time of this study; age at first symptom and at first tumor; number and type of tumors; and total sun exposure as indicated by the patient himself^5^. The median number of epithelioma per patient was 41, 15, and 1 for aggressive, medium, and mild symptoms, respectively^5^. These cohorts are described in Tables 1 (French cohort) and 2 (Brazilian cohort).

#### French cohort (Cohort 1)

We previously analyzed a retrospective cohort of 23 XP-V patients (21–85 years old) from unrelated families in terms of clinical, molecular and genetic data^5^. Their median age at clinical XP-V diagnosis was 22 years and the median age of skin cancer occurrence was 21 years. The genetic analysis of mutations on the *POLH* gene was correlated to the severity of the disease^5^. We had the opportunity to obtain new blood samples from 11 among these 23 patients and 10 non XP-V controls, ranging from 23 to 85 years of age (Table 1). The 11 patients were originated from France (5 patients), North Africa (1 from Tunisia, and 2 from Algeria), Turkey (1 patient), Kosovo (1 patient) and Congo (1 patient). All these patients live in France and are followed in French university hospitals. Interestingly, among these patients, we were able to obtain blood samples from three of them 10 years after the first time they were studied, which allowed us to make an individual follow-up of the evolution of their SHM profile in two patients with a complete loss of Pol η and in one patient with only a partial expression of this protein. The three French patients that were reanalyzed 10 years later are indicated in Table 1.

**Table 1:** Description of the *POLH* mutations of patients from cohort 1 (France).

XP-V Patients	Age at blood sampling	*POLH* mutation	Amino acid change	Severity of symptoms
XP961VI	42	c.437 dupA	p.Tyr146Ter	Mild
XP965VI	23	c.907 C>T	p.(Arg303*)	Aggressive
XP966VI	85	c.108_110del3	p.(Val37del)	Medium
XP967VI	71	c.108_110del3	p.(Val37del)	Mild
XP968VI	32	c.672delA c.-5+1G>C	p.(Lys224Trpfs*229) p.?	Mild
XP888VI	35	c.672delA c.-5+1G>C	p.(Lys224Trpfs*229) p.?	Mild
XP422VI	54 and 64	c.907 C>T c. 1222_1225delACTT	p.(Arg303*) p. (Thr408LeufsX36)	Medium
XP603VI	55 and 65	c.883 G>A c.1727_8 delCT	p.(Gly295Arg) p.(Pro576Argfs*3)	Aggressive
XP127VI	56	c.1093dupC	p.(Gln365Profs*27)	Aggressive
XP737VI	42	c.-5+1 G>C	p.?	Mild
XP606VI	33 and 43	c.-5+1 G>C	p.?	Medium
Controls				
XP964VI (XP-E)	41			
XP1001VI (XP-C)	36			
M1	35			
M2	29			
M3	25			
M4	39			
O1	79			
O2	84			
O3	73			
O4	80			

#### Brazilian cohort (Cohort 2)

Blood samples were collected from an isolated Brazilian XP-V cohort of 12 patients, ranging from 13 to 79 years of age, recently described^23^, as well as from 12 non XP-V controls belonging to the same community, including five patient relatives (bearing monoallelic *POLH* mutations). So far, 17 XP-V patients have been described in the region, in a population of approximately 7,000 individuals. The 12 patients studied here live in the same community of approximately 220 inhabitants. Despite the high incidence of the disease, the clinical diagnosis of the first patient (XP06GO) was only reported in 2009. The majority of the population has Caucasian origin, even though some families have black ancestry.

In contrast to the French cohort, which is non-homogeneous in terms of patient origins and *POLH* mutations, the second cohort comes from a community located at the same geographical region in the center of Brazil, with patients living together in close “consanguinity”. Therefore, this cohort is very homogenous with only two different *POLH* mutations which originate from two distinct founder events: one at the splice donor site of intron 6 (c.764 + 1 G > A), and the other in exon 8 (c. 907 C > T, p. Arg 303*). The characteristics of the 12 XP-V patients, as well as the controls from the same community published recently^23^, are summarized in Table 2.

**Table 2:** Description of the *POLH* mutations of patients from cohort 2 (Brazil).

XP-V Patient	Age at blood sampling	Genomic mutation	Amino acid change	Severity of symptoms
XP52GO	44	c.764+1 G>A	splice donor variant	Aggressive
XP39GO	39	c.764+1 G>A c.907 C>T	splice donor variant c.907 C>T [p.(Arg303*)]	Aggressive
XP06GO	13	c.764+1 G>A	splice donor variant	Mild
XP03GO	36	c.764+1 G>A c.907 C>T	splice donor variant c.907 C>T [p.(Arg303*)]	Aggressive
XP88GO	79	c.907 C>T	c.907 C>T [p.(Arg303*)]	Mild
XP11GO	64	c.764+1 G>A	splice donor variant	Mild
XP04GO	40	c.764+1 G>A c.907 C>T	splice donor variant c.907 C>T [p.(Arg303*)]	Aggressive
XP110GO	17	c.764+1 G>A	splice donor variant	Mild
XP85GO	77	c.764+1 G>A	splice donor variant	Aggressive
XP33GO	33	c.764+1 G>A	splice donor variant	Mild
XP63GO	32	c.764+1 G>A c.907 C>T	splice donor variant c.907 C>T [p.(Arg303*)]	Aggressive
XP56GO	35	c.764+1 G>A c.907 C>T	splice donor variant c.907 C>T [p.(Arg303*)]	Mild
Controls				
GO119^#^	62			
GO46^#^	14			
GO05^#^	37			
GO01^#^	39			
GO113	58			
GO40	75			
GO89^#^	56			
GO129	64			
GO07	58			
GO126	34			
GO128	69			
GO127	56			

^#^Heterozygous

### SHM pattern correlates with Pol η activity

**Figure 1:**
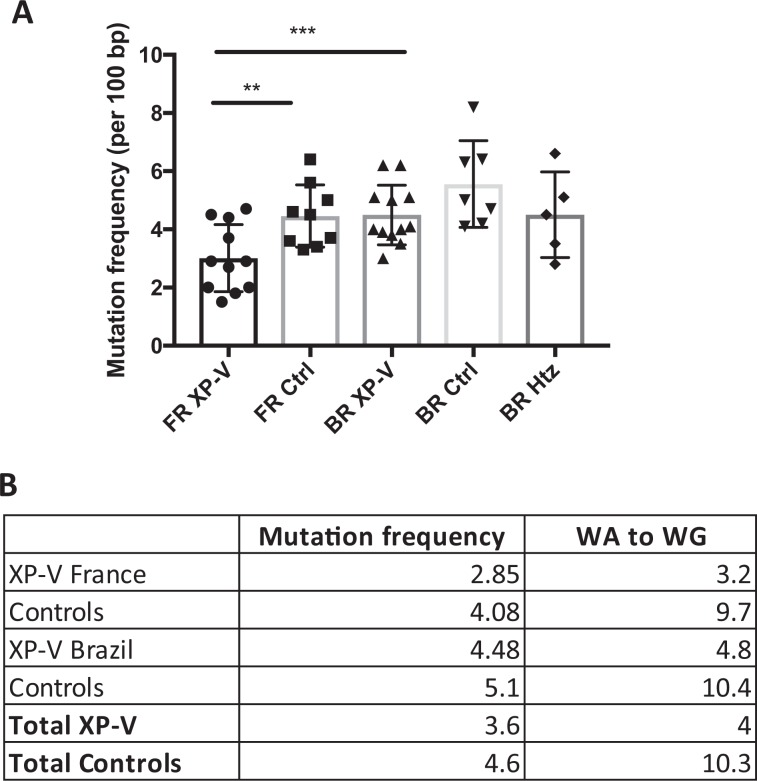
Mutation frequency is lower in XP-V patients than in controls. (**A**) Mutation frequency (per 100 bp) of XP-V patients (FR XP-V) and controls (FR Ctrl) of the French cohort, as well as XP-V patients (BR XP-V), wild-type (*POLH* homozygous) (BR Ctrl) and carrier (*POLH* heterozygous) (BR Htz) controls from the Brazilian cohort. **p = 0.01 and ***p = 0.0037 (Unpaired T-test). (**B**) SHM mutation frequency (per 100 bp), percentages of transitions and transversions and mutations in WA to WG motifs of XP-V patients, carriers and the corresponding controls.

Analysis of Ig SHM was performed on DNA isolated from blood memory B cells. The mutation spectrum (% GC: AT) and Pol η protein expression detected by western blot are reported in Supplementary Table 1 (French cohort), in Supplementary Table 2 (Brazilian cohort) and in Supplementary Table 3 (both cohorts combined). The analysis showed that the mutation frequency was in average 4.4/100 bp for the controls and 3/100 bp for the French XP-V patients (Fig. 1A) indicating that the XP-V patients undergo significantly less point mutations than healthy individuals. However, the analysis of Ig SHM of the Brazilian cohort showed different results compared to the French cohort in terms of mutation frequency. Although both the XP-V and the heterozygous carriers display lower mutation frequency relative to the controls, the overall mutation frequency is not significantly different in all groups (Fig. 1A,B), showing in average 5.1 mutations per 100 bp for the wild-type (*POLH* homozygous) controls, 4.5/100 bp for the carrier (*POLH* heterozygous) controls, and 4.5/100 for the XP-V patients, which strikingly differs from the XP-V patients of the French cohort. Interestingly, we observed a significant difference in mutation frequency between the XP-V patients from the two cohorts, the mutation frequency of the Brazilian XP-V patients being significantly higher (Fig. 1A).

**Figure 2:**
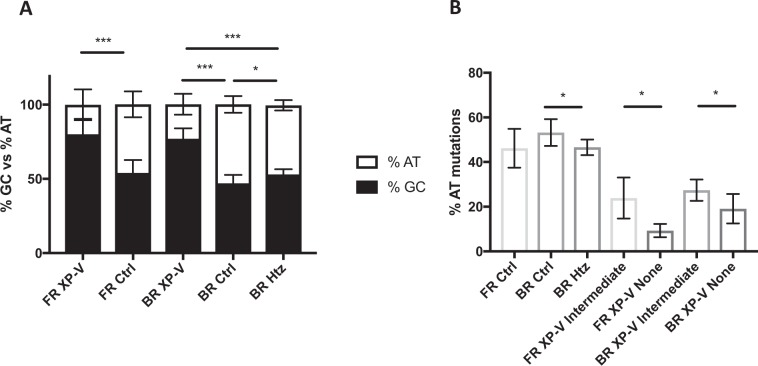
Percentage of AT mutations is proportional to DNA polymerase η activity. (**A**) SHM profile as percentage of AT: GC mutations of XP-V patients (FR XP-V) and controls (FR Ctrl) of the French cohort, as well as XP-V patients (BR XP-V), wild-type (*POLH* homozygous)(BR Ctrl) and carrier (*POLH* heterozygous)(BR Htz) controls from the Brazilian cohort. *p<0.05  and ***p<0.0001 (Unpaired T-test). (**B**) Percentage of AT mutations of *POLH* WT controls from the French (FR Ctrl) and Brazilian (BR Ctrl) cohorts, *POLH* heterozygous (carrier) controls from the Brazilian cohort (BR Htz), XP-V patients with an intermediate pol η activity of the French (FR XP-V Intermediate) and Brazilian (BR XP-V Intermediate) cohort, and XP-V patients with no detectable pol η activity from the French (FR XP-V None) and Brazilian (BR XP-V None) cohorts. *p = 0.0418 (Unpaired T-test).

In accordance with previous observations^21,22^, we found a drastic reduction of mutations on A/T bases in the French XP-V patients compared to the controls (19.9% *vs*. 42.2%), as well as in the Brazilian XP-V patients compared to both the wild-type (23.3% *vs*. 53.2%) and the carrier (23.3% *vs*. 46.6%) controls (Fig. 2A, Supplementary Fig. S2A,B, and Supplementary Tables 1–3). Importantly, for both cohorts, the overall A/T transversion mutations (WA to WG) that corresponds to the mutagenic signature of Pol η are reduced in the XP-V patients compared to the control groups (Fig. 1B and Supplementary Figs. S1B and S2).

Furthermore, our data show a direct correlation between the level of expression of active Pol η and the rate of A/T mutations at the Ig JH4 intron. As illustrated in Fig. 2B, the percentage of A/T mutations were analyzed in B cells from (i) healthy individuals (*POLH* WT, controls), (II) heterozygous controls (*POLH* Htz), (iii) XP-V patients with intermediate levels of Pol η (detectable by WB) (XP-V Intermediate), and (vi) XP-V patients with no Pol η (none). For the French cohort, compared to the A/T frequency of 49.7% observed in *POLH* WT healthy individuals and 46.6% in *POLH* heterozygous healthy individuals, patients (both cohorts combined) with residual Pol η present an intermediate A/T mutation frequency of 24.1%, whereas in those with no Pol η the A/T frequency drops to 15.5% (Supplementary Table 3). Similar results were found in the Brazilian cohort. Strikingly, we observed a significant difference in the SHM profile between the WT (*POLH* homozygous) individuals and the carrier’s (*POLH* heterozygous) population (Fig. 2B). To our knowledge, this is the first time that *POLH* heterozygous are reported to display a decrease in A/T mutations in Ig genes, indicating that, at least for this Brazilian community, *POLH* is haploinsufficient for the process of SHM. Together, the findings from both cohorts expand and corroborate the major role of DNA Pol η in A > G and T > C transitions during SHM even in conditions where the latter is present in reduced quantity (Supplementary Table 3).

### Pol η deficiency leads to an increased frequency of deletions in Ig genes that correlates with patient age

**Figure 3:**
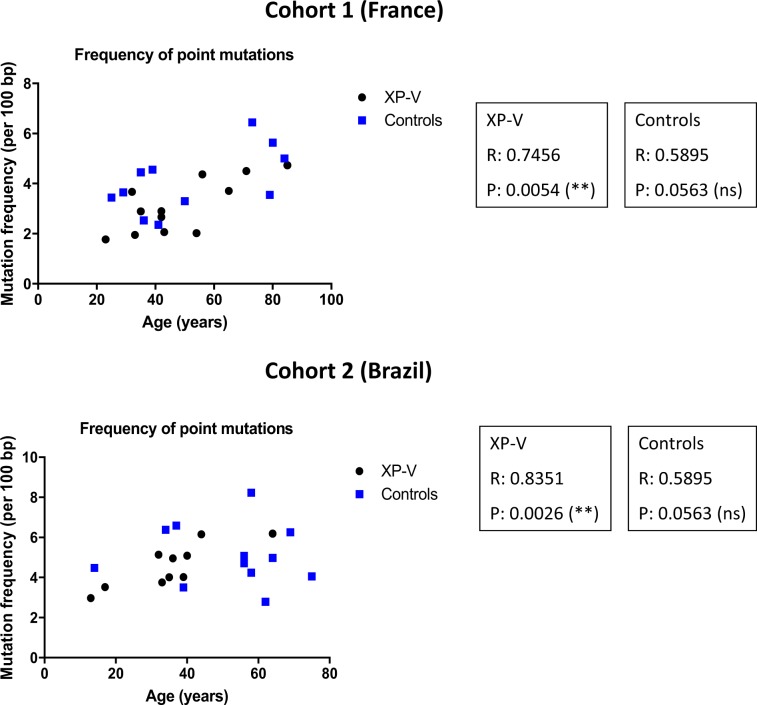
Correlation between frequency of point mutations and age in XP-V patients. Frequency of point mutation (per 100 bp) was plotted against age (years) of XP-V patients and controls of the French (upper panel) and Brazilian (bottom panel) cohorts. Values of Pearson correlation (R) and p values are indicated for each group (XP-V and controls) of both cohorts.

Pol η has also been reported to have access to undamaged DNA during several cellular processes^24–27^. However, the best characterized Pol η activity on non-damaged DNA remains its role in A/T mutagenesis during SHM in B cells. Therefore, the mechanism of SHM offers a unique opportunity to evaluate with accuracy the consequences of Pol η deficiency and its long-term substitution by other TLS DNA polymerases on genome stability maintenance and possibly in the emergence of new mutational signatures. To address this question, we analyzed the SHM pattern of the mutated Ig from young and elderly patients. Besides the change in the pattern of A/T and G/C mutations, the overall point mutation frequency increases in an age-dependent manner (Fig. 3), indicating an accumulation of mutations in the Ig gene throughout the life of the memory B cells. This increase is highly significant in XP-V patients from both cohorts.

**Figure 4:**
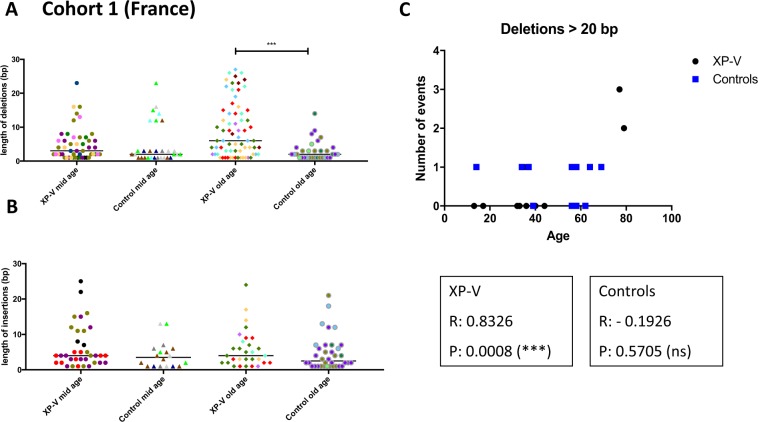
XP-V patients over 50 years of age present higher frequency of large deletions (larger than 10 bp) compared to age-matched controls. (**A**) Size of deletions (in base pairs) of XP-V patients (older than 50 years) and controls of the French cohort. Mid age: younger than 50 years. Old age: older than 50 years. ***p < or = 0.0005 (1-way Anova test). Each color represents one patient/control. (**B**) Size of insertions of XP-V patients (older than 50 years) and controls of the French cohort. Each color represents one patient/control. (**C**) Number of large deletions (larger than 20 bp) was plotted against age (years) of XP-V patients and controls of the Brazilian cohort. Values of Pearson correlation (R) and p values are indicated for each group (XP-V and controls).

A detailed analysis of the other mutational events occurring during the process of SHM at Ig heavy chain in the French cohort showed a significant increase of large deletions (>10 bp) in patients older than 50 years compared to the control groups (Fig. 4A and Supplementary Table 4). For the insertions however, it should be noted that even though the average insertion size is greater in XP-V patient cells, it remains not significantly different from those of controls (Fig. 4B). Similar to the French cohort, we found a significant accumulation of large deletions (>20 bp) with age in the Brazilian XP-V patients, which does not exist for the control population, and a significant increase of larger deletions (>20 bp) in patients older than 50 years compared to the control group in the Brazilian cohort (Fig. 4C and Supplementary Fig. S3A). It is noteworthy that there was no significant difference when we used a cut off of 10 bp used for the French cohort (Supplementary Table 5). Comparably to the French cohort, the average insertion size in XP-V patient cells from the Brazilian cohort exhibits a slight increase in the old XP-V patients compared to the other groups (Supplemenatry Fig. S3B).

**Figure 5:**
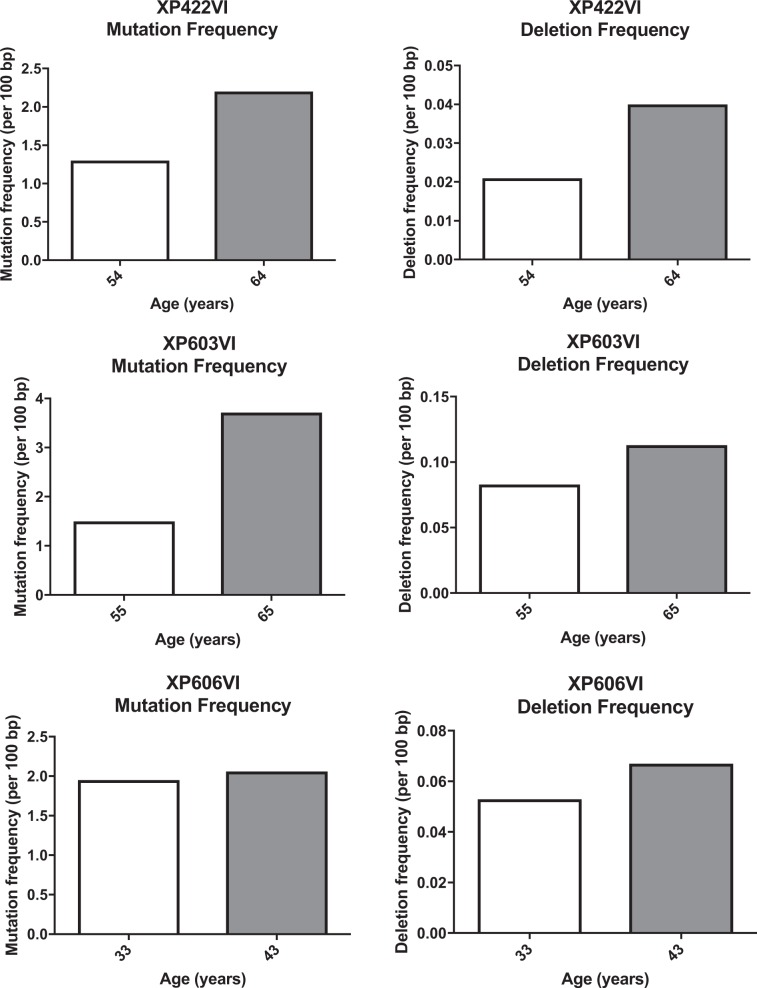
10 year follow up of 3 XP-V patients show increased mutation and deletion frequencies. Mutation and deletion frequencies (per 100 bp) of the three XP-V patients from the French cohort reanalyzed 10 years later, showing values for the first and the second samplings.

To further validate this observation, and to address the question of time-related accumulation of indels in Ig genes in the absence of Pol η, we analyzed the pattern of SHM from three French XP-V patients that we have described 12 years ago^22^ (Table 1). We found that, for both older patients (XP422VI and XP603VI), from whom the two blood samples were collected after the age of 50 years, the frequency of both mutations and indels (mainly deletions) had greatly increased over the 10-year period (Fig. 5)^28^. For the third patient (XP606VI), whose blood samples were collected at the age of 33 and 43 years, the analysis result showed only a small increase in both mutation and indel frequencies. Together, these data indicate that at least in the case of Ig genes, the sustained absence of Pol η leads, in XP-V patients aged 50 years and older, to profound sequence alterations probably due to its substitution by other TLS polymerases.

### Analysis of SHM pattern for XP-V diagnosis

One possible translational objective is to routinely use in genetic counseling the SHM analysis as a standard, straightforward, quick and reliable assay for XP-V syndrome diagnosis. Having these two large cohorts, we thought to validate this approach. Five mL of blood were collected from suspected XP-V patients (individuals with sun sensitivity and who develop skin tumors on exposed sites) and the genomic DNA purified from isolated memory B cells was used for both *POLH* sequencing and the immunoglobulin J_H_4 intron amplification and sequencing. As proof of concept, SHM pattern was established in a blind approach to determine the diagnosis before sequencing the *POLH* gene. In 100% of cases, the analysis of the pattern of SHM gave the right diagnosis which was subsequently confirmed by *POLH* sequencing. Interestingly, we analyzed the SHM pattern of two patients who were clinically diagnosed as potential XP-V and found no alteration of the pattern of mutations at Ig locus, and the sequencing of *POLH* confirmed the absence of mutation. The sequencing of other XP genes showed a mutation in *XPC* for one patient and in *XPE* for the other, which confirms the robustness and reliability of the test (Supplementary Tables 1 and 4).

## Discussion

Somatic hypermutation is a base substitution process that targets the variable regions of Ig genes during high-affinity antibody acquisition. Strikingly, in the antigen-activated B cells, the DNA repair mechanisms are used in a rather paradoxical way in order to promote mutagenesis, by recruiting error-prone DNA polymerases. Accumulated data and overwhelming evidence show that Pol η is involved in the mutagenesis of Ig genes by converting the WA motif to WG (W: A/T) by preferentially incorporating dGTP opposite the T template, this pattern allowing to assign to Pol η a specific mutational signature on undamaged template. However, in addition of its function in SHM, Pol η has been shown to access undamaged DNA in many other cellular contexts, including replication of Common Fragile Sites (CFS), recombination intermediates, maintenance of telomeric DNA, replication of cohesin-bound DNA and, recently, its participation in the synthesis of lagging strand during yeast DNA replication^24–27^. A Pol η mutational signature that matches the spectrum of Pol η has been identified in solid tumors associated to UV exposure and alcohol consumption^29^. Therefore, in the absence of Pol η and thanks to its substitution by other TLS polymerases, major alterations should be expected in the previously described Pol η-dependent DNA transactions.

In this report, we focus on the mechanism of SHM in order to investigate the impact of a constitutive Pol η deficiency on the Ig gene mutation pattern for two reasons: (1) Pol η recruited in activated B cells acts as a gap-filling polymerase on undamaged templates, and (2) Among all other DNA polymerases present in the B cell, Pol η is selectively chosen, by unknown mechanisms, as the only A/T bp “mutator” during the normal process of SHM. However, its absence opens a window of opportunities for the other DNA TLS polymerases usually excluded from the process to be recruited. Therefore, the mechanism of SMH offers an unique physiological model that can be used to address the consequences of sustained Pol η deficiency and its substitution by other polymerases not only in terms of genome stability but also to anticipate its consequences in other loci in the genome of B and non-B cells where its activity is reported to be important^24–27^.

By analyzing both the non-homogeneous French cohort, which includes patients with many different *POLH* mutations, and the very homogeneous Brazilian cohort (only two different *POLH* mutations), we demonstrated the strict correlation between Pol η activity and the percentage of A/T mutations in Ig genes. We show that the patient genotypes that abolish Pol η expression or/and activity reduce A/T mutations up to 80 to 90%, while patient genotypes that either destabilize the mRNA, increase the turnover of the protein, or moderately affect the activity of the enzyme and lead to a reduction of A/T mutations proportionally to its expression/activity. Notably, the significant difference in the GC: AT mutations between the wild-type controls and the heterozygous carriers in the Brazilian cohort corroborates the strict correlation between Pol η activity and the percentage of A/T mutations in Ig genes. To our knowledge, this is the first time that a significant difference in SHM profile between *POLH* wild-type and heterozygous individuals is reported.

Since several procedural as well as technical issues have precluded the development of routine diagnostic services for XP patients, the results from this report and our previously published results^28^, together with data from other laboratories^30^, indicate with high confidence that the analysis of SHM pattern can be used as a reliable, quick and low cost assay in clinic, from a small volume of blood, not only for the XP-V diagnosis (to differentiate it from other XP complementation groups) but also for estimation of the presence of any residual activity of Pol η. Indeed, in the French cohort, the western blot analysis and estimation of Pol η of the XP-V patient XP603VI that displays an amino acid substitution (Gly295Arg) show that the corresponding polypeptide runs at the WT full length size and the expression level was classified as intermediate (Supplementary Table 1). This level of expression is similar to two other patients, XP966VI and XP967VI, who express a mutated form of Pol η (p.Val37del). However, the analysis of SHM indicates that, while the expressed enzyme in XP966VI and XP967VI exhibits a clear DNA polymerase activity since we observed only a moderate decrease in A/T mutations due to its low level expression, the mutation spectrum of XP603VI, on the contrary, resembles that of Pol η-deficient XP-V patients, indicating that the expressed polypeptide is inactive. Therefore, a simple analysis of SHM indicates that the deletion of Val37 has no major impact on Pol η activity, while the substitution of Gly 295 by Arg abrogates its activity. A recent structural model of Pol η showed that G295R is, indeed, very disruptive to the protein structure^5^.

Interestingly, while the partial or complete absence of Pol η in B cells does not lead to any major apparent immunological defect due to joint action of G/C mutations and the affinity selection process, in terms of disease development, all the XP-V patients were prone to skin cancer development. However, we found no strict correlation between complete or partial expression of Pol η protein and the severity of the disease. This might be an indication that, in conditions of reduced availability of Pol η in the skin, depending on the sun exposure, only a fraction of the UV-induced DNA lesions are bypassed in an error-free manner, while the remaining lesions are processed in an error-prone fashion by other TLS polymerases, leading to an increase of both mutation load and increased predisposition for skin cancer development. In activated B cells, the acquisition of the “best” mutations due to B cell affinity selection compensates for the absence or low abundance of Pol η and B cell aging.

A striking result from our current study is that, in the absence of Pol η, an increase in the proportion of deletions in the J_H_4 intron sequence of Ig genes in memory B cells is observed, and this pattern becomes more visible with age. Memory B cells are generated during the germinal center (GC) reaction in the course of T cell-dependent immune responses and are characterized by increased lifespan, faster response to antigen stimulation, and expression of somatically mutated high affinity Ig genes. These long lived cells can be reactivated rapidly as many times as necessary upon reencounter of the cognate antigen, proliferate and either differentiate into plasma cells to provide immediately specific and high affinity antibodies, and/or re-start the GC reaction to undergo new rounds of affinity maturation to adapt to the pathogen changes. In these ways, the memory B cell receptor (BCR) is re-exposed to AID-driven new rounds of proliferation and mutagenesis^31–34^.

Because memory B cell clones can persist in human for decades^35,36^, we assumed that the older the patient, the longer the memory cell has had to re-enter several times the GC and undergo several rounds of SHM. Therefore, the consequences of Pol η deficiency on the Ig locus can be easily seen, especially in aged patients since their memory B cells had a longer period of time to enter several cycles of SHM and to accumulate mutations/deletions. When we analyzed the pattern of SHM of French XP-V patients, we found that the frequency of large deletions (>10 bp) increases significantly, in an age-dependent manner (mainly after 50 years of age), compared to age-matched healthy individuals. To further confirm this observation, we analyzed the J_H_4 intron of patients from the homogenous cohort from Brazil, and obtained similar results with an increased number of large deletions (>20 bp) compared to age-matched individuals from the same family/community. Finally, we analyzed the pattern of SHM in three XP-V patients twice at ten years apart. These results indicate a clear increase of deletion events, when compared to the levels ten years earlier, only in XP-V patients older than 50 years. Collectively, our data indicate that in the absence of Pol η, a substitute TLS polymerase(s) participate(s) in the establishment of a new mutation landscape.

At this stage, we can only speculate on the candidates. Based on the expression profile of different DNA polymerases in GC B cells available in the Immgen database (immgen.org), we found that few of them are induced in GC B cells: Rev1 and REV3L are induced 2- to 3-fold compared to naive B cells, while Pol η and Pol θ are more strongly induced, 6- and 15-fold respectively (Supplementary Fig. 5). Although all these enzymes seem to be good candidates to substitute Pol η during both SHM in B cells and other DNA transactions in the other cells, only Pol θ specific activities could partially fit with the observed pattern. Indeed, in the mouse, Pol θ was shown to participate in end-joining (EJ) of a DNA break during Ig class-switching, producing insertions of base pairs at the joins with homology to IgH switch-region sequences^37^. Furthermore, in *D. melanogaster*, by analysis of the repair of transposase-induced DNA double strand breaks (DSBs), a specific role for Pol θ in Alternative-EJ was proposed^38^. Pol θ-mediated end-joining mutational signature, which consists on the presence of large deletions flanking the break site, was also identified in embryonic stem cells^39^. Finally, recent work by the Prakash group showed a collaboration between Pol θ and Pol η to prevent replication fork collapse and, similarly to the mechanism of recruitment of Pol η to UV-damaged DNA and during SHM, PCNA ubiquitylation is required for the assembly of Pol θ into foci in UV-damaged human cells^40^. These results point to a possible role of Pol θ in promoting localized deletions when repairing DNA DSBs, although further investigations are needed to confirm the role of Pol θ in indel generation. The current data highlights the consequences of the activity of backup polymerases that act in the absence of Pol η in terms of both mutation occurrence, pattern modification, and likely genome instability.

## Methods

### Patients

The French patients analyzed in this study belong to a cohort of XP-V patients diagnosed in our laboratory in the last 20 years. The major clinical and molecular data of these patients have been recently published^5^. The Brazilian patients have been clinically described and their cellular and molecular characterization has been published recently^23^. Full patient informed consent for study participation and sequencing of V(D)J genes has been obtained at the time of blood removal for both cohorts. For participants under the age of 18 years, informed consent was obtained from a parent and/or legal guardian. Approval of the studies was given by the French Agency of Biomedicine (Paris, France) for the French part and the Ethical Committee for Human Research of the Institute of Biomedical Sciences, University of Sao Paulo (Sao Paulo, Brazil) for the Brazilian part. All methods were performed in accordance with the relevant guidelines and regulations of the appropriate institutions (University of Sao Paulo, Sao Paulo, Brazil and Gustave Roussy, Villejuif, France).

### B cell isolation

Human memory B cells were isolated from 3–10 mL of blood samples, collected in vacuum tubes with heparin. The blood was diluted in PBS/EDTA buffer (PBS pH 7.2, 2 mM EDTA) and centrifuged at 400 g for 30 minutes at 20 °C in a swinging-bucket rotor without brake, after carefully adding twice the amount of Ficoll (Ficoll-Paque PLUS, GE Healthcare Lifesciences, Little Chalfond, UK). After formation of the density gradient, the layer containing the peripheral blood mononuclear cells (PBMCs) was carefully collected, washed with PBS/EDTA/BSA buffer (PBS pH 7.2, 2 mM EDTA, 0.5% BSA). CD19^+^ CD27^+^ IgD^−^ memory B cells were purified by cell sorting as previously described^41^. DNA extraction was performed in 200 μL of PBS and 20 μL of Proteinase K (20 mg/mL, Macherey-Nigel, Duren, Germany) at 56 °C for 60 minutes, followed by 10 minutes at 95 °C.

### Analysis of mutations

For the determination of the pattern of mutations in human V_H_ genes, we used a set of six primers to amplify all human V_H_ genes together with a J_H_5 primer, in order to amplify the J_H_4 intronic sequence (340 bp) as described^42^. The PCR was performed on aliquots of 4,000–6,000 cells with a high-fidelity Polymerase (Phusion High-Fidelity DNA Polymerase, ThermoFischer Scientific, Waltham, USA), and four to five PCR reactions were pooled to avoid biases in the analysis. Pooled amplified sequences were then cloned using the pCR4 Blunt TOPO Ligation Kit (Invitrogen, ThermoFischer Scientific), transformed in chemically competent *E.coli* bacteria (One Shot Top10 chemically competent *E. coli*, Life Technologies, ThermoFischer Scientific) and plated in kanamycin selective medium. Colonies containing the insert were sequenced by GATC Biotech (Konstanz, Germany). A mean of 30 mutated sequences (±5) and/or 200 mutations (±20) were analyzed per individual (Supplementary Table 6). Sequences were aligned with the free software Codon Code Aligner (available for download at http://www.codoncode.com/aligner/), and the comparative frequency analysis was done with SHM Tool^43^. To calculate mutation frequency, the accumulated number of unique mutations was divided by the theoretical maximum number of the corresponding type of mutation, to correct for base composition^43^. Only distinct and independent clones were used in the analysis.

## Supplementary information

Supplementary data.
