# Supplementary material for: Large deletions in immunoglobulin genes are associated with a sustained absence of DNA Polymerase η
Source: Sci Rep. 2020 Jan 28;10:1311. doi: 10.1038/s41598-020-58180-7 (PMC6987143; doi:10.1038/s41598-020-58180-7)
Supplement: Supplementary file 1 — Supplementary data. [file 41598_2020_58180_MOESM1_ESM.pdf]

Large deletions in immunoglobulin genes are associated with a sustained absence of DNA Polymerase η

Leticia K. Lerner[1,2,5], Thuy V. Nguyen[1,6], Ligia P. Castro[2], Juliana B. Vilar[2], Veridiana Munford[2], Morwenna Le Guillou[1,4], Mahwish Mian Mohammad[1,4], Véronique Vergé[3,4], Filippo Rosselli[1,4], Carlos F.M. Menck[2], Alain Sarasin[1,4], Said Aoufouchi[1,4*].

[1]Centre National de la Recherche Scientifique UMR8200, Gustave Roussy, 94805 Villejuif, France

[2] Department of Microbiology, Institute of Biomedical Sciences, University of São Paulo, São Paulo, Brazil

[3] Haematology Unit, Gustave Roussy, 94805 Villejuif, France

[4] Université Paris-Saclay, 91400 Orsay, France

[5] Current address: Medical Research Council Laboratory of Molecular Biology, Cambridge, UK

[6] Current address: Department of Genetics, Faculty of Biology, University of Science, Ho Chi Minh City, Vietnam

[*] Corresponding author: Said Aoufouchi, CNRS UMR8200, Gustave Roussy, 94805, Villejuif, France. E-mail: said.aoufouchi@gustaveroussy.fr

Supplementary Tables

Supplementary Table 1: Pol η expression and mutation spectrum of cohort 1 (France)

XP-V Patient	Pol η expression *	Mutation spectrum GC:AT	Transitions:transversions
XP961VI	None	88.4:11.6	52.9:46.1
XP965VI	None	94.2:5.8	55.7:44.3
XP966VI	Intermediate	65.2:34.8	39.4:60.6
XP967VI	Intermediate	62.4:37.6	38.7:61.3
XP968VI	Intermediate	79.2:20.8	52.7:47.5
XP888VI	Intermediate	78.1:21.9	47.2:52.8
XP422VI	None	89.4:10.6	51.7:48.3
		88.1:11.9 §	57.3:42.7 §
XP603VI	Intermediate	89.5:10.5	53.4:46.6
		82.8:17.2 §	53.7:46.3 §
XP127VI	Intermediate	85.4:14.6	46.1:53.9
XP737VI	Intermediate	73.5:26.5	55.0:45.0
XP606VI	Intermediate	75.8:24.2	53.0:47.0
		67.2:32.8 §	47.2:52.8 §
**Controls**	**Pol η expression ***	**Mutation spectrum GC:AT**	**Transitions:transversions**
M1	Normal	52.8:47.2	57.4:42.6
M2	Normal	60.3:39.7	54.9:45.1
M3	Normal	56.3:43.7	56.1:43.9
M4	Normal	43.6:56.4	47.7:52.3
O1	Normal	67.8:32.2	59.3:40.7
O2	Normal	62.6:37.4	59.8:40.2
O3	Normal	46.4:53.6	44.0:56.0
O4	Normal	43.0:57.0	47.5:52.5
**Others**	**Pol η expression ***	**Mutation spectrum GC:AT**	**Transitions:transversions**
XP964VI (XP-E)	Normal	51.5:45.5	57.8:42.2
XP1001VI (XP-C)	Normal	51.6:48.4	51.7:48.3

* estimated by Western Blot

§ 10 years later

Supplementary Table 2: Pol η expression and mutation spectrum of cohort 2 (Brazil)

XP-V Patient	Pol η expression *	Mutation spectrum GC:AT	Transitions:transversions
XP52GO	Intermediate	85.0:15.0	49.0:51.0
XP39GO	None	75.6:24.4	46.3:53.7
XP06GO	Intermediate	79.7:20.3	54.2:45.8
XP03GO	None	75.8:24.2	41.1:58.9
XP88GO	N/A	65.5:34.5	50.0:50.0
XP11GO	Intermediate	69.4:30.6	37.7:62.3
XP04GO	None	73.8:26.2	44.6:55.4
XP110GO	Intermediate	73.3:26.7	55.9:44.1
XP85GO	Intermediate	74.2:25.8	52.6:47.4
XP33GO	Intermediate	73.5:26.5	50.9:49.1
XP63GO	None	84.9:15.1	53.3:46.7
XP56GO	None	90.0:10.0	49.6:50.4
**Controls**	**Pol η expression ***	**Mutation spectrum GC:AT**	**Transitions:transversions**
GO119	Normal	58.1:41.9	45.9:54.1
GO46	Normal	49.4:50.6	48.1:51.9
GO05	Normal	54.6:45.4	49.8:50.2
GO01	Normal	50.7:49.3	62.7:37.3
GO113	Normal	44.7:55.3	54.1:45.9
GO40	Normal	52.4:47.6	44.5:55.5
GO89	Normal	54.3:45.7	49.4:50.6
GO129	Normal	39.1:60.9	48.5:51.5
GO07	Normal	51.6:48.4	48.8:51.2
GO128	Normal	53.1:46.9	56.3:43.7
GO127	Normal	45.0:55.0	49.5:50.5
GO126	Normal	41.5:58.5	47.4:52.6

* estimated by Western Blot

N/A: fibroblasts not available

Supplementary Table 3: Pol η expression and mutation spectrum of both cohorts combined

XP-V Patient	Pol η expression *	Mutation spectrum GC:AT	Severity of symptoms
XP961VI	None	88.4:11.6	Mild
XP965VI	None	94.2:5.8	Aggressive
XP422VI	None	89.4:10.6	Medium
		88.1:11.9 §	
XP39GO	None	75.6:24.4	Aggressive
XP03GO	None	75.8:24.2	Aggressive
XP04GO	None	73.8:26.2	Aggressive
XP63GO	None	84.9:15.1	Aggressive
XP56GO	None	90.0:10.0	Mild
XP966VI	Intermediate	65.2:34.8	Medium
XP967VI	Intermediate	62.4:37.6	Mild
XP968VI	Intermediate	79.2:20.8	Mild
XP888VI	Intermediate	78.1:21.9	Mild
XP603VI	Intermediate	89.5:10.5	Aggressive
		82.8:17.2 §	
XP127VI	Intermediate	85.4:14.6	Aggressive
XP737VI	Intermediate	73.5:26.5	Mild
XP606VI	Intermediate	75.8:24.2	Medium
		67.2:32.8 §	
XP52GO	Intermediate	85.0:15.0	Aggressive
XP06GO	Intermediate	79.7:20.3	Mild
XP11GO	Intermediate	69.4:30.6	Mild
XP110GO	Intermediate	73.3:26.7	Mild
XP85GO	Intermediate	74.2:25.8	Aggressive
XP33GO	Intermediate	73.5:26.5	Mild
**Average XP-V no pol η expression**		84.5:15.5	
**Average XP-V intermediate pol η expression**		75.9:24.1	
**Average controls**		51.4:48.6	

* estimated by Western Blot

§ 10 years later

Supplementary Table 4: Pol η expression, frequency of point mutations and indels of cohort 1 (France)

XP-V Patient or Control	Age at blood sampling	Pol η expression	Mutation frequency*	Indel frequency*	Deletions £	Insertions £
XP965VI	23	None	1.77	0.025	3 < 10	1 < 10
XP961VI	42	None	2.66	0.07	6 < 10	none
XP422VI	54	None	2.02	0.021	2 < 10, 3 > 20	none
XP422VI §	64	None	1.3	0.04	3 < 10, 1 > 10 < 20	1 < 10, 1 > 10 < 20
XP968VI	32	Intermediate	3.67	0.22	10 < 10	10 < 10, 2 > 10 < 20
XP606VI	33	Intermediate	1.95	0.053	2 < 10, 1 > 20	1 < 10, 2 > 20
XP888VI	35	Intermediate	2.89	0.402	13 < 10, 3 > 10 < 20	5 < 10, 5 > 10 < 20
XP737VI	42	Intermediate	2.9	0.157	6 < 10, 1 > 10 < 20	8 < 10
XP606VI §	43	Intermediate	2.06	0.067	4 < 10, 1 > 10 < 20	1 < 10
XP603VI	55	Intermediate	1.5	0.119	3 < 10, 3 > 10 < 20, 4 > 20	3 < 10
XP127VI	56	Intermediate	4.37	0.163	6 < 10, 2 > 10 < 20, 2 > 20	none
XP603VI §	65	Intermediate	3.71	0.083	11 < 10, 2 > 10 < 20, 1 > 20	2 < 10, 2 > 10 < 20
XP967VI	71	Intermediate	4.5	0.15	7 < 10, 5 > 10 < 20, 1 > 20	7 < 10
XP966VI	85	Intermediate	4.73	0.308	10 < 10, 2 > 10 < 20, 1 > 20	12 < 10, 1 > 10 < 20, 1 > 20
M3	25	Normal	3.44	0.091	2 < 10	3 < 10
M2	29	Normal	3.65	0.147	6 < 10, 1 > 10 < 20	2 < 10, 1 > 10 < 20
M1	35	Normal	4.45	0.117	4 < 10	2 < 10
XP1001VI (XP-C)	36	Normal	2.53	0.031	1 < 10, 2 > 10 < 20	none
M4	39	Normal	4.56	0.343	6 < 10, 1 > 10 < 20	7 < 10
XP964VI (XP-E)	41	Normal	2.36	0.055	4 < 10, 2 > 10 < 20, 1 > 20	2 < 10, 1 > 10 < 20
O3	73	Normal	6.44	0.508	9 < 10	9 < 10, 1 > 20
O1	79	Normal	3.55	0.196	4 < 10	2 < 10, 3 > 10 < 20
O4	80	Normal	5.64	0.533	11 < 10	17 < 10, 1 > 10 < 20
O2	84	Normal	5	0.09	3 < 10	1 < 10

§ 10 years later

* per 100 bp

£ number and size (in bp)

Supplementary Table 5: Pol η expression, frequency of point mutations and indels of cohort 2 (Brazil)

XP-V Patient or Control	Age at blood sampling	Pol η expression	Mutation frequency*	Indel frequency*	Deletions £	Insertions £
XP63GO	32	None	5.14	0.226	2 < 10, 4 > 10 < 20	4 < 10
XP56GO	35	None	4.01	0.183	9 < 10, 1 > 20	none
XP03GO	36	None	4.95	0.316	12 < 10	2 < 10
XP39GO	39	None	4.02	0.274	6 < 10, 1 > 10 < 20	6 < 10, 1 > 10 < 20
XP04GO	40	None	5.09	0.245	9 < 10, 1 > 10 < 20	none
XP06GO	13	Intermediate	2.97	0.123	6 < 10, 2 > 10 < 20	none
XP110GO	17	Intermediate	3.52	0.126	7 < 10, 1 > 10 < 20	1 > 10 < 20
XP33GO	33	Intermediate	3.75	0.262	14 < 10, 4 > 10 < 20	7 < 10
XP52GO	44	Intermediate	6.15	0.343	7 < 10, 3 > 10 < 20	3 < 10, 1 > 10 < 20
XP11GO	64	Intermediate	6.19	0.514	12 < 10, 1 > 10 < 20, 1 > 20	12 < 10, 2 > 10 < 20
XP85GO	77	Intermediate	3.93	0.777	18 < 10, 1 > 10 < 20, 3 > 20	14 < 10, 1 > 10 < 20
XP88GO	79	N/A	4.06	0.294	2 < 10, 2 > 20	2 < 10, 5 > 10 < 20
GO46	14	Normal	4.48	0.311	20 < 10, 1 > 20	13 < 10, 2 > 20
GO126	34	Normal	6.38	0.407	3 < 10, 2 > 10 < 20, 1 > 20	11 < 10, 1 > 20
GO05	37	Normal	6.59	0.294	10 < 10, 1 > 10 < 20, 1 > 20	none
GO01	39	Normal	3.5	0.061	None	3 < 10, 1 > 10 < 20
GO89	56	Normal	5.08	0.294	8 < 10, 1 > 20	3 < 10
GO127	56	Normal	4.71	0.138	3 < 10, 1 > 10 < 20	4 < 10, 1 > 10 < 20
GO07	58	Normal	8.23	0.504	5 < 10, 1 > 10 < 20	5 < 10, 1 > 20
GO113	58	Normal	4.24	0.26	10 < 10, 4 > 10 < 20	13 < 10, 3 > 10 < 20
GO119	62	Normal	2.79	0.093	4 < 10, 2 > 10 < 20	1 < 10
GO129	64	Normal	4.98	0.226	5 < 10, 1 > 20	4 < 10
GO128	69	Normal	6.25	0.122	4 < 10	1 > 20
GO40	75	Normal	4.05	0.484	15 < 10, 2 > 10 < 20, 2 > 20	8 < 10, 1 > 10 < 20

* per 100 bp

£ number and size (in bp)

N/A: non available

Supplementary Table 6: Sequencing depth for each group of Cohorts 1 (France) and 2 (Brazil)

	XP-V France	Control France	XP-V Brazil	Control Brazil
Number of individuals	11	9	12	12
Number of mutated sequences	342	150	187	223
Total mutations	4,669	1,813	2,084	3,246
Total bp sequenced	116,740	58,290	64,600	47,750

Supplementary Figures legends

Supplementary Figure 1. A**:** Age is similar between all XP-V patients and control groups of this study. Ages (years) of XP-V patients (FR XP-V) and controls (FR Ctrl) of the French cohort, as well as XP-V patients (BR XP-V), wild-type (*POLH* homozygous) (BR Ctrl) and carrier (*POLH* heterozygous) (BR Htz) controls from the Brazilian cohort. B: SHM profile as percentage of AT: GC mutations of XP-V patients (FR XP-V) and controls (FR Ctrl) of the French cohort, as well as XP-V patients (BR XP-V), wild-type (*POLH* homozygous) (BR Ctrl) and carrier (*POLH* heterozygous) (BR Htz) controls from the Brazilian cohort as in Figure 2A, but displaying the values for transversions and transitions in A/T templates.

Supplementary Figure 2. A**:** Mutation pattern in rearranged J_H4 intronic sequences from XP-V patients and controls of the French cohort. B: Mutations in rearranged J_H4 intronic sequences from XP-V patients, wild-type (*POLH* homozygous) and carrier (*POLH* heterozygous) controls from the Brazilian cohort. Data are corrected for base composition. Values are expressed as number of mutations. n, represents the total number of base substitutions determined for each genotype.

Supplementary Figure 3: XP-V patients over 50 years old present higher frequency of large deletions (larger than 20 bp) than age-matched controls. A: Size of deletions (in bp) of XP-V patients older than 50 years and controls of the Brazilian cohort. Mid age: younger than 50 years. Old age: older than 50 years. B: Size of insertions of XP-V patients (older than 50 years) and controls of the Brazilian cohort.

Supplementary Figure 4: DNA polymerase θ is highly expressed in germinal center B cells. Expression levels for different polymerases (Polh: η; Rev1, Polq: θ; Polk: Pol κ; Poli: Pol ι; Rev3L: catalytic subunit of Pol ξ) from the Immgen database were plotted as ratios between germinal center B cells and splenic B cells (GC/S ratio).

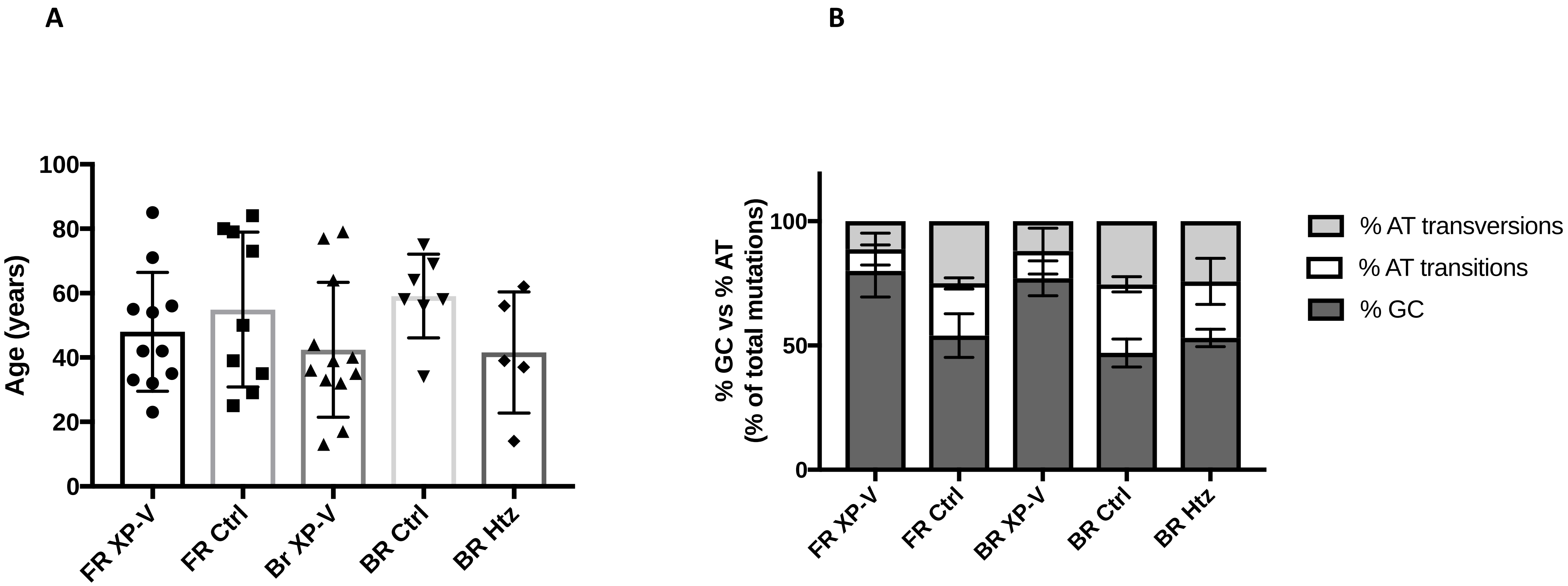

Supplementary Figure 1

A Cohort 1 (France)

Controls

From \ To	G	C	A	T	Total
G	0	152	284	38	474
C	131	0	66	261	458
A	170	174	0	80	424
T	89	210	108	0	407

932 G/C (n = 1763)

XP-V

From \ To	G	C	A	T	Total
G	0	451	705	140	1296
C	533	0	279	663	1745
A	143	142	0	128	313
T	132	26	137	0	295

3041 G/C (n = 3648)

B Cohort 2 (Brazil)

Controls

From \ To	G	C	A	T	Total
G	0	139	205	57	401
C	173	0	76	203	452
A	219	149	0	90	458
T	135	294	94	0	523

853 G/C (n = 1834)

Carriers

From \ To	G	C	A	T	Total
G	0	118	205	46	369
C	130	0	67	173	370
A	128	95	0	67	291
T	83	204	96	0	383

739 G/C (n = 1413)

XP-V

From \ To	G	C	A	T	Total
G	0	23	461	112	596
C	449	0	205	496	1150
A	140	1	0	87	228
T	124	4	56	0	184

1746 G/C (n = 2158)

Supplementary Figure 2

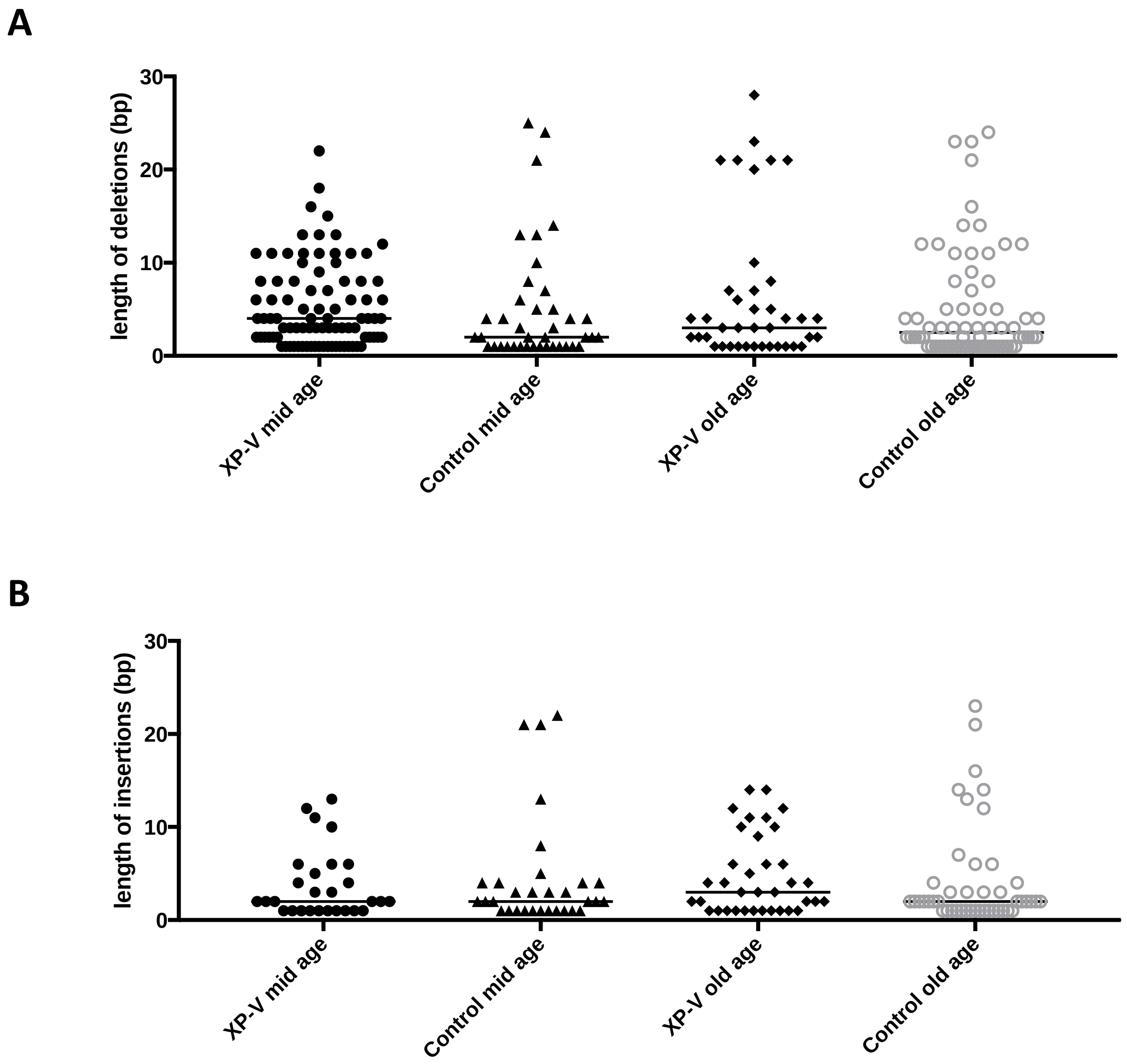

Supplementary Figure 3

Ratio Expression in GC B cell/ Expression in splenic B cell

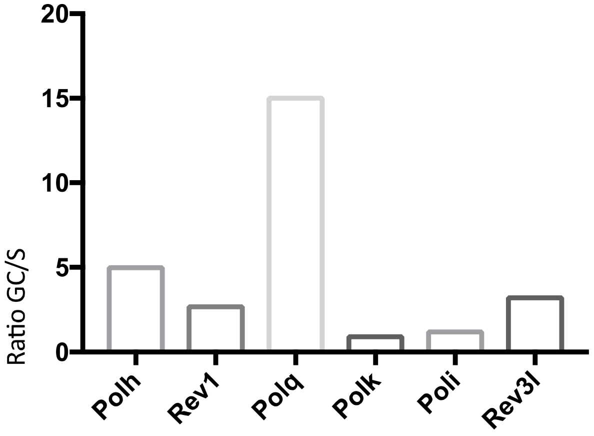

Supplementary Figure 4
